# Supplementary material for: Technology-Based Substance Use Interventions: Opportunities for Gender-Transformative Health Promotion
Source: Int J Environ Res Public Health. 2020 Feb 5;17(3):992. doi: 10.3390/ijerph17030992 (PMC7037203; doi:10.3390/ijerph17030992)
Supplement: Supplementary file 1 [file ijerph-17-00992-s001.pdf]

Table S1. Included Studies.

**Brief web-based interventions**	**Study design**	**Research Aim**	**Population**	**Intervention/Procedure (excludes control conditions)**	**Key Outcomes**	**Authors' Assessment of Gender Inclusion Level Based on Gender Continuum (see Figure 1)**
Choo et al., 2016 [28]	Randomized control trial	Assess the acceptability and feasibility of BSAFER: Brief Intervention for Substance Use and Partner Abuse for Females in the Emergency Room	n= 40 women with coexisting experiences of intimate partner violence and substance use disorders.	BSAFER, a brief web-based intervention conducted on a tablet during an emergency department visit that included goal-setting questions/videos with a supplemental phone booster 2 weeks later.	Small decrease in weekly substance use (0.7 days); 95% completed all portions of the BSAFER web program. Readiness for substance use change increased at 2-weeks (1.4 points intervention group compared to 0.5 points control group on 5-point scale).	Gender transformative: addresses the causes of gender inequities (violence against women and co-occurring substance use disorder) and includes ways to transform harmful norms and relations (empowerment, goal setting, social supports).
Fang et al., 2010 [26]	Randomized control trial	Examine the efficacy and generalizability of a family-oriented, web-based substance use prevention program to young Asian-American adolescent girls.	n=108 mother-daughter dyads comprised of Asian girls aged 11-14 and their mothers.	Nine-session web-based substance use prevention program aiming to prevent girls' substance use by enhancing mother-daughter interactions.	Improved self-efficacy and refusal skills, reduced depressed mood, higher levels of mother-daughter closeness and communication, fewer instances of	Gender transformative: addresses health and gender inequities from an intersectional approach, incorporating problem-solving

					substance use, and lower intentions to use in the future.	skills and self-efficacy, using a health promotion approach to body image and substance use.
Gilmore et al.,2016 [30]	Randomized control trial (secondary analysis)	Examine the effectiveness of a web-based combined sexual assault risk and alcohol use reduction intervention on anxiety outcomes.	n= 264 college women who engaged in heavy episodic drinking	One-time web-based combined alcohol use and sexual assault risk reduction intervention using personalized normative feedback.	Decreased drinking to cope with anxiety among women with stronger drinking to cope motives in the intervention group compared to control.	Gender sensitive: the program incorporates sexual assault risk reduction to substance use programming, and secondary analysis examines data based on gender. However, details of gender-specific inequities and if/how they are addressed are lacking.
Gilmore et al., 2015 [29]	Randomized control trial	Assess the effectiveness of a web-based combined sexual assault risk and alcohol use reduction intervention.	n= 207 college women who engaged in heavy episodic drinking	One-time web-based combined alcohol use and sexual assault risk reduction intervention using personalized normative feedback.	Women with higher severity of sexual assault engaged in less binge drinking in the intervention compared to control.	Gender sensitive: the program incorporates sexual assault risk reduction to substance use programming. However, considerations of gender-specific needs and ways in

						which harmful norms will be transformed are lacking.
Gryczynski et al., 2018 [31]	Randomized control trial (secondary analysis)	Examine gender and ethnicity differences in uptake of a computerized vs. in-person brief intervention for substance misuse among adult primary care patients.	n= 359 adult primary care patients with moderate-risk illicit substance use (45% women, 55% men).	Self-directed, computer-delivered brief motivational interviewing intervention (up to two sessions) that include gender-specific normative feedback in regards to substance use.	Hispanic men responded more favourably to computer-based brief intervention compared to in-person, with the reverse being true for Hispanic women.	Gender-sensitive: gender-specific normative feedback was used in the intervention, but details of how gender-specific needs are being addressed is lacking.
Koski-Jannes et al., 2007 [32]	Longitudinal study	Evaluate an internet-based alcohol self-assessment tool for Finnish individuals who drink.	n= 924 Finnish individuals who drink (54.9% women, 45.1% men)	Brief (5-minute) internet-based self-assessment with psychosocial and alcohol use questions, followed by personalized normative feedback based on responses.	Women were more inclined to use self-assessment compared to men. There were no gender differences in drinking-related outcomes.	Gender sensitive: the intervention alludes to sex-specific personal normative feedback measures, but transformative details are lacking.
Lewis et al., 2007 [35]	Randomized control trial	Evaluate whether gender-specific referents influence the efficacy of a personalized normative feedback brief intervention for alcohol consumption.	n= 185 college students (45.2% women, 54.8% men)	Gender-specific, or gender-neutral personalized normative feedback surveys.	Gender specific normative feedback reduced drinking behaviour for women only but not for men. The gender-specific intervention was most effective for women who self-	Gender sensitive: the intervention includes gender-specific personalized normative feedback, but measures to accommodate gender-specific

					identified most strongly to their gender.	needs are not presented.
Lewis et al., 2007 [33]	Randomized control trial	Evaluate the efficacy of a computerized personalized normative feedback intervention on reducing alcohol consumption among high risk college freshmen.	n= 316 high risk drinking college freshmen (53.8% women, 46.2% men).	Computerized brief intervention gender-specific and freshmen-specific personalized normative feedback.	Norm perceptions of typical same-gender drinking was positively associated with riskier drinking behaviour compared to opposite gender typical norm perceptions. Participants in the gender-specific intervention group showed greater drinking reductions compared to control.	Gender sensitive: the intervention includes gender-specific personalized normative feedback, but measures to accommodate gender-specific needs are not presented.
Schinke et al., 2011 [23]	Randomized control trial	Test a computer-delivered family intervention approach for preventing substance use with African American and Hispanic American adolescent girls.	n= 546 mother-daughter dyads comprised of Black and Hispanic American-identified adolescent girls and their mothers.	Home-based 10 session internet or CD-ROM gender-specific substance use prevention program guided by family interaction theory, completed by mother-daughter dyads. Dyads were advised to complete one 45 min. session/week.	Intervention group showed reduced risk factors, improved protective factors, reduced alcohol use, fewer intentions to use substances in the future among girls, and better levels of communication	Gender transformative: addresses health and gender inequities, incorporating problem-solving skills and self-efficacy, using a health promotion approach to body esteem, mental

					between mothers and daughters.	health and substance use.
Schinke et al., 2009 [22]	Randomized control trial	Evaluate a gender-specific computer-based intervention to prevent underage drinking among early adolescent girls.	n= 202 mother-daughter dyads (girls aged 10-13 years).	Home-based 14-session internet or CD-ROM gender-specific substance use prevention program guided by family interaction theory, completed by mother-daughter dyads. Dyads completed between 4-5 sessions/week, email reminders 2x per week.	Intervention group showed improved communication between mothers and daughters. Follow up showed improved alcohol refusal skills, healthier normative beliefs about underage drinking, and reduced alcohol consumption, and intentions to drink in the future among girls.	Gender transformative: addresses health and gender inequities, incorporating self-efficacy, refusal skills, social relationships, and a health promotion approach to gender-specific needs.
Schinke et al., 2009 [25]	Randomized control trial	Evaluate a gender-specific computer-based intervention to prevent substance use among adolescent girls at 2-year follow up.	n= 916 mother-daughter dyads (girls aged 11-13 years).	Home-based 9-session internet or CD-ROM gender-specific substance use prevention program guided by family interaction theory, completed by mother-daughter dyads. Dyads were advised to complete 1 45min. session/week.Refer to Schinke, Fang & Cole (2009).	Girls in intervention group reported fewer risk factors, stronger protective factors, and less substance use.	Gender transformative: addresses health and gender inequities, incorporating problem-solving skills and self-efficacy, using a health promotion approach to body esteem, mental health and substance use.

Schinke et al., 2009 [24]	Randomized control trial	Evaluate a gender-specific computer-based intervention to prevent substance use among adolescent girls at 1-year follow up.	n= 591 mother-daughter dyads (girls aged 11-13 years).	Refer to Schinke et al., 2009 [23]	Compared to control, girls in intervention group reported reduced substance use, stronger refusal skills, lower intentions to use substances in the future, and stronger mother-daughter communication.	Gender transformative: addresses health and gender inequities, incorporating problem-solving skills and self-efficacy, using a health promotion approach to body esteem, mental health and substance use.
Schwinn et al., 2014 [21]	Randomized control trial	Evaluate a gender-specific computer-based intervention to prevent substance use among early adolescent girls who reside in public housing.	n= 36 mother-daughter dyads living in publicly subsidized housing (girls aged 10-12 years).	Three-session web-based health promotion program for mother-daughter dyads guided by family interaction theory. One session/week for 3 weeks with email/telephone call reminders.	Intervention group showed stronger mother-daughter communication, stronger substance use refusal skills and reduced stress among girls.	Gender transformative: addresses health and gender inequities, incorporating problem-solving skills and self-efficacy, using a health promotion approach to body esteem, mental health and substance use.
Schwinn et al., 2016 [48]	Descriptive	Describe the process and challenges associated with developing and programming a	N/A	RealTeen 9-session narrator-guided web-based substance misuse prevention program incorporating gender-	N/A	Gender-specific: the intervention acknowledges gender norms and acts to

		gender-specific web-based intervention to prevent substance use among adolescent girls.		specific risk factors for girls. One session/week, 15-20 minutes each.		accommodate these needs to some degree through goal setting and coping methods. However, some aspects of the intervention further instill gender stereotypes i.e. those about beauty
Schwinn et al., 2010 [27]	Randomized control trial	Evaluate a gender-specific Internet-based intervention to prevent substance use among early adolescent girls.	n= 236 girls in grade 7—9	RealTeen 12-session narrator-guided web-based substance misuse prevention program incorporating gender-specific risk factors for girls. Two sessions/week, 25 minutes each.	Girls in the intervention group showed reduced substance use at 6-month follow up and developed more positive normative beliefs and greater increased self-efficacy.	Gender-specific: the intervention acknowledges gender norms and acts to accommodate these needs to some degree through goal setting and coping methods. However, some of the intervention further instill gender stereotypes i.e. those about beauty
Thompson et al., 2018 [34]	Randomized control trial	Evaluate the impact of a brief web-based intervention on drinking outcomes and perceived norms among first-year university students.	n= 245 first year university students living in residence (66% women, 34% men).	One-time online self-administered personalized normative feedback alcohol intervention.	Intervention group showed lower norm misperceptions about peer drinking. There were no gender-based differences in perceptions.	Gender blind: no gender-based aspects are described in the intervention.

Computer games	Study design	Research Aim	Population	Intervention/procedure (excludes control conditions)	Key Outcomes	Authors' Assessment of Gender Inclusion Level Based on Gender Continuum (see Figure 1)
Verduin et al., 2013 [45]	Randomized control trial	Examine the impact of a computer videogame simulation on relapse prevention skills for individuals with alcohol use disorder.	n= 41 veteran men in outpatient substance use treatment	Guardian Angel computer videogame simulation program to enhance alcohol relapse prevention skills by guiding a character through decision making. Eight sessions within 12 weeks.	Intervention group showed reduced binge drinking and higher rates of self-efficacy.	Gender blind: though the study has presented results of a gender group, the intervention presents no details on gender-based norms, relations and inequities.
Wetter et al., 2011 [46]	Randomized control trial	Examine the effectiveness of a palmtop computer-delivered treatment for smoking relapse prevention among women.	n= 302 women who smoke tobacco	Palmtop computer-delivered smoking relapse prevention treatment following counseling (from day 7-day 35 post-counseling) including information on managing urges, motivational messaging and treatment information.	The intervention was most effective for women who were heavier smokers. The intervention did not improve cessation relative to the control group.	Gender sensitive: the intervention alludes to including one aspect of gender-specific design (coping skills in relation to depression), although overall gender-based considerations in lacking.
Rundle-Thiele et al., 2013 [43]	Pre-/post-survey	Examine the feasibility of a social marketing and education-based intervention game for	n= 223 Year 10 Australian students (n=131 boys in all-boys	Game On:Know Alcohol (GO:KA) 6-module (4.5 hours) intervention for	Intention to change (reduce drinking) among for girls but not for boys.	Gender blind: no gender-based considerations are

		moderating teen alcohol intake.	school; n=102 girls in all-girls school, tested separately)	moderating teen alcohol intake, including game and knowledge-based sessions in connection to drinking behaviours and attitudes.	Attitude change was also more positive for girls.	described in the intervention.
**Mobile phone application (app)**	**Study design**	**Research Aim**	**Population**	**Intervention/procedure (excludes control conditions)**	**Key Outcomes**	**Authors' Assessment of Gender Inclusion Level Based on Gender Continuum (see Figure 1)**
Abrantes et al., 2017 [54]	Descriptive	Describe the development of a lifestyle Fitbit physical activity app for women with comorbid alcohol dependence and depression.	n= 20 women with depression in partial hospital-based treatment for alcohol misuse	12-week health promotion Fitbit intervention for alcohol harm reduction, relapse prevention, and depression, incorporating physical activity. 6x weeks 30-minute phone counseling sessions. Thirty-minute phone calls took place at weeks 1, 2, 4, 6, 8, and 10 of the intervention.	Participants attended 78% of counseling sessions and wore the Fitbit for 73% of treatment days. 75% completed treatment intervention; among women who completed the intervention, 44% remained abstinent from alcohol throughout treatment. Over the course of treatment, participants reported an increase in using physical	Gender transformative: intervention takes an intersectional health promotion approach to improve health and address gender-based harms in relation to substance use and mental health co-morbidities.

					activity to cope with urges to drink and to cope with negative affect.	
Antoine et al., 2016 [52]	Cross sectional survey	To examine interest in technology-assisted substance use disorder treatment.	174 participants in outpatient treatment for substance use disorder (43% women, 57% men).	21-item survey investigating interest in technology-assisted substance use disorder treatment.	Compared to men, women showed a higher preference for computer-based substance use disorder treatment.	N/A (not examining a specific intervention)
Baskerville et al., 2016 [50]	Qualitative descriptive study (focus group)	Examine LGBTQ+ youth and young adults' perceptions of a culturally tailored mHealth app for smoking cessation.	n= 204 LGBTQ+ youth and young adults aged 16-29 years (39% women).	Twenty-four focus groups to determine perceptions of a culturally tailor mHealth app for smoking cessation.	Participants preferred a culturally tailored mHealth app for smoking cessation due to its accessibility, ability to connect with community, personalization, and monitoring and tracking. Key concerns were related to privacy, access, and transparency.	N/A (intervention is not in full development)
Gajecki et al., 2014 [19]	Randomized control trial	Examine the efficiency of two smartphone apps targeting drinking choices on party occasions, with the goal of reducing problematic	n= 1,932 Swedish university students (51.7% women, 48.3% men)	Two interventions: mHealth apps targeting drinking planning and choices in order to reduce problematic drinking. Promillekoll	Significantly more men did not complete follow up in the PartyPlanner group compared to the women. Men in	Gender blind: though a sex-based measure was used for blood alcohol content level, and the study secondary

		alcohol intake among Swedish university students.		app: government developed publicly-downloadable app where user can check their blood alcohol concentration (BAC). Party Planner app: user can check/compare BAC before and after drinking event, and input alcohol consumption plans. Seven-week trial with 1 email use reminder.	the Promillekoll group showed increased drinking frequency.	analyses examined gender differences, the intervention was lacking of any gender-specific norms, relations and inequities.
Gordon et al., 2017 [51]	Pre-/post-test	Develop and test the feasibility of the See Me Smoke-Free mHealth app to address smoking and health promotion among women who smoke.	n= 151 women who smoke tobacco	See Me Smoke-Free health promotion mHealth app to address smoking and track healthy lifestyle habits. Ninety- day user pilot.	47% of participants reported 7-day tobacco abstinence at 90 days, and significant increases in physical activity and fruit consumption. Participants reported moderate levels of satisfaction with the app. Attrition was high at 52%.	Gender transformative: the intervention acknowledges gender-specific smoking cessation concerns, and offers gender-transformative solutions encompassing health promotion and economic status.
Kim et al., 2014 [53]	Cross sectional survey (secondary analysis)	Examine whether patient preferences for technology-based interventions varies by gender.	n= 417 patients in the emergency department (45.1% men, 54.9% women)	iPad survey assessing preferences of technology-based vs. non-technology-based health information and	Women were more likely to use computers, Internet, and social networks compared to men.	N/A (details of specific interventions not provided)

				support in connection to risky behaviours including substance misuse.	Men and women preferred technology-based formats for health information but were concerned about confidentiality. Women of older age showed decreased interest in technology-based information for smoking cessation (not the case for men).	
Leonard et al., 2017 [49]	Mixed methods	Evaluate the acceptability and feasibility of the Mind the Moment (MtM) intervention for women college students who engage in problem drinking.	n= 10 women college students (non-treatment seeking and undergraduate)	Two in-person brief alcohol intervention counseling sessions + the use of the MtM wearable sensor band/mobile app (incorporating motivational interviewing and CBT techniques) for approx. 3-4 weeks.	High levels of acceptability of the MtM intervention, promoted increased awareness related to environmental stressors and drinking behaviours. Functionality issues of sensor band were reported.	Gender-specific: the intervention takes a health promotion approach and identifies a need for a women-specific approach to alcohol problem drinking, addressing some specific needs.
**Social media, websites and online communities**	**Study design**	**Research Aim**	**Population**	**Intervention/procedure**	**Key Outcomes**	**Authors' Assessment of Gender Inclusion Level Based on**

						Gender Continuum (see Figure 1)
Bottorff et al., 2014 [38]	Cross sectional survey	Explore youth-informed perspectives of gender-specific video messaging on tobacco exposure and its connections to breast cancer and its potential to be used on social media.	n= 135 adolescent boys and girls (54% girls, 46% boys).	Brief survey assessing two youth-informed gender-specific videos to potentially be used on social media, designed to raise awareness among adolescents about tobacco exposure and its connections to breast cancer.	The majority of girls strongly agreed or agreed that they learned something new from the girls' video messaging and that the video contained important information for teens. The same was true for boys' reaction to the boy-specific video.	Gender specific: the program identifies sex and gender-based health concerns, and addresses the need to accommodate to some degree.
Branley et al., 2017 [44]	Cross sectional survey	Examine if exposure to social media content depicting risky behaviour predicts users' own behaviour offline, and if this correlation is stronger for men.	n= 412 young adults aged between 18 and 25 years (71.1% women, 28.9% men) who use social media.	One-time online survey assessing connections between risky behavior (including substance use) being depicted online, and an individual's personal risky behaviour.	There is a relationship between exposure to online content depicting risky substance use behaviour and offline behaviour. There were no gender moderating effects.	N/A (not examining a specific intervention)
Cantrell et al., 2016 [36]	Longitudinal study	Examine the utilization of BecomeAnEx.org smoking cessation website among young adults and young adult subgroups	n= 5,983 new registered users on a smoking cessation website aged 18 – 70 years (61.5% women, 38.5% men).	BecomeAnEX.org smoking cessation website usage trackage for 6 months. Website include quit plans, separation exercises	Young adults aged 18 to 34 years visited and used the site features less than adults 35 and older. Compared to women, men were	Gender blind: no gender-based considerations are described in the intervention.

		compared with older adults to identify patterns by age, gender, race, and ethnicity.		and ways to avoid triggers.	significantly less likely to use the website, particularly among young adults.	
Haines-Saah et al., 2015 [40]	Qualitative descriptive study (prospective non-comparative)	Determine the feasibility of a tobacco reduction cessation intervention for young adults that uses digital photography and social networking.	n= 60 young adults aged 16-24 who currently smoke cigarettes or quit within the last year (57% men, 43% women).	12-week online participant-driven photography group on Facebook for self-identified current cigarette smokers to share daily activities and participate in photo challenges in connection to smoking cessation.	Women were more active with posting compared to men. Both men and women indicated their overall experience with the intervention was positive and would participate in the project again.	Gender blind: no gender-based considerations are described in the intervention.
Larm et al., 2017 [39]	Cross sectional survey	Examine whether online social network chatting is related to peer-related pathways to alcohol use among adolescents, including stress exposure, peer status, and social context pathways.	n= 2,439 boys and girls aged 15-16 years enrolled in the 9th grade of Swedish primary school (51.7% girls, 48.3% boys).	One-time questionnaire evaluating online social network chatting and its potential connections to peer-related pathways to alcohol use.	Online social network chatting had a positive association on alcohol use reduction for boys and girls. The association between social context peer-related pathways to alcohol use was stronger among girls compared to boys.	N/A (not examining a specific intervention)
Laz et al., 2013 [62]	Cross sectional survey	Examine the influence of race and ethnicity on seeking health	n= 3,181 women aged 16–24 years	Self-administered survey examining women's internet use to	More Caucasian and African American women	N/A (not examining a specific intervention)

		information from the internet among women aged 16-24 years.		access health information.	used the internet to locate health information than Hispanic women (highest use among Caucasian women). African American women were significantly less likely to seek information on general health concerns compared to Caucasian women. This was not true for Hispanic women.	
Lygidakis et al., 2010 [63]	Randomized control trial	Assess the feasibility of collecting health behaviour survey data from a web-based survey.	n= 190 high school students aged 14-16 years (50.5% boys, 49.5% girls)	Web-based survey regarding lifestyle, alcohol use, and tobacco use to report health behaviour data and feasibility.	Boys who filled out the web-based survey reported higher per hour drink consumption and more binge drinking compared to those who filled out the paper-based survey. Girls who filled out the web-based survey reported younger age of onset for alcohol use, and were more likely to report being	N/A (not examining a specific intervention)

					monthly smokers compared to the paper-based survey.	
Smith et al., 2011 [37]	Cross sectional survey	Examine current opinions of smoking cessation efforts and their socioeconomic and political interconnections in the context of the recession.	n= 150 employees representative of major financial and corporate firms in the rustbelt	42-item survey examining smoking patterns, socioeconomic and political perspectives on smoking cessation efforts.	Men in particular find technology-based social networking and virtual communities to be beneficial to smoking cessation.	N/A (not examining a specific intervention)
**Telephone interventions**	**Study design**	**Research Aim**	**Population**	**Intervention/procedure (excludes control conditions)**	**Key Outcomes**	**Authors' Assessment of Gender Inclusion Level Based on Gender Continuum (see Figure 1)**
Abraham et al., 2017 [58]	Qualitative descriptive study (focus group)	Assess the feasibility and acceptability of a collaborative telephone-delivered intervention for promotion alcohol behaviour change among women veterans with unhealthy drinking and co-morbid depression and/or probable PTSD.	n= 19 women veterans with unhealthy drinking and co-morbid depression and/or probable post-traumatic stress disorder.	Focus group assessing feasibility of 60-minute collaborative telephone-delivered alcohol intervention for women veterans with co-morbid depression, potential PTSD, and alcohol misuse. Emphasis on choice, options, and collaborative planning/goal setting.	Participants perceived the length of time as a potential barrier feasibility and acceptability was favourable.	Gender specific: the intervention incorporates shared decision-making with an intersectional group, providing some accommodation, but does lack focus/detail on solutions to transforming gender-based norms and relations.

Kim et al., 2016 [61]	Randomized control trial	Examine the acceptability and feasibility of a videoconferencing smoking cessation intervention for Korean American women compared to a telephone-based intervention.	n= 49 Korean American women who smoke and were interested in smoking cessation.	Eight 30-minute weekly individual counseling sessions focused on cultural connections to smoking cessation (by phone or videoconference) and nicotine patches for 8 weeks.	The telephone intervention was preferred for women over 50 years old. Self-reported abstinence was higher for the video conferencing intervention.	Gender transformative: the intervention was developed in order to minimize barriers created by intersectional gender-based stigma and work to address social, cultural, and environmental issues that contribute to smoking and barriers to accessing smoking cessation programs.
Levine et al., 2013 [12]	Cross sectional survey	Compare weight gain concerns among tobacco cessation quitline callers of different weight classifications.	n= 595 callers to a tobacco quitline (66.1% women, 33.9% men).	Survey assessing weight-related concerns, motivation to quit smoking, and overall tobacco use among tobacco quitline callers.	Compared to men, significantly more women endorsed postcessation weight gain concerns.	N/A (details of specific interventions not provided)
McKay et al., 2011 [59]	Randomized control trial	Determine whether 18 months of telephone continuing care improves outcomes for patients with alcohol dependence.	n= 252 alcohol-dependent continuing care patients (64.3% men, 35.7% women).	Two interventions: brief (5-10 minute) telephone calls for up to 18 months, offered weekly for the first 8 weeks, followed by every other week for the next 10 months for	Women showed significant effects in favour of both telephone-based interventions compared to treatment as usual.	Gender blind: no gender-based considerations are described in the intervention.

				continuing care patients looking to reduce risky alcohol use. Each call consisted of a 10-item assessment of alcohol behaviour and related risk/protective factors. Progress tracked quarterly for 24 months. The second intervention was the same treatment in addition to counseling.		
Rasmussen et al., 2013 [60]	Ecological study	Assess the cost-effectiveness of a Danish smoking cessation telephone service quitline.	n= 2,758 smoking quitline callers (57.4% women, 42.6% men)	Danish smoking cessation telephone quitline. Free of charge service operating 24 hours/week.	The majority of callers were women with an average age of 40.9.	N/A (details of specific interventions not provided)
**Targeted web-based messaging**	**Study design**	**Research Aim**	**Population**	**Intervention/procedure (excludes control conditions)**	**Key Outcomes**	**Authors' Assessment of Gender Inclusion Level Based on Gender Continuum (see Figure 1)**
Richardson et al., 2013 [41]	Randomized control trial	Evaluate feasibility and acceptability of web-based messages describing the relationship between secondhand smoke and breast cancer among boys and girls.	n= 1,498 students aged 13 – 15 years (44.22% boys, 55.78% boys).	Web-based survey with tailored web-based gender- and culturally-specific about the relationship between second hand smoke and breast cancer. After presentation of	Girls in intervention group were 14% more likely to agree that exposure to secondhand smoke increased their breast cancer risk and were 52% more likely to	Gender specific: the program identifies intersectional gender-based health concerns, and addresses the need to accommodate to some degree.

				messages, user-specific questions were asked on smoking tobacco behaviour and attitudes.	request additional information on these topics. Boys in the intervention group were 10% more likely to agree that girls' exposure to secondhand smoke increased their breast cancer risk.	
Schwartz et al., 2014 [42]	Randomized control trial	Evaluate the efficacy of targeted gender-specific web-based messages about the increased risk of breast cancer associated with cigarette smoke exposure.	n= 618 non-smoking girls aged 13 – 15 years.	Targeted gender and culturally-specific messages delivered online that presented risk connections between breast cancer and cigarette smoke.	Girls in the intervention group were significantly more likely to identify breast cancer being caused by cigarette smoke, and to agree that secondhand smoke exposure increased their risk of breast cancer.	Gender specific: the program identifies intersectional gender-based health concerns, and addresses the need to accommodate to some degree.
Bottorff et al., 2014 [47]	Qualitative descriptive study (focus group)	Use a focus group to guide design of gender and cultural-specific messaging in regards to the link between tobacco exposure and breast cancer.	n= 43 First Nations, Métis and non-Indigenous boys and girls between the ages of 12 and 17 (58% boys, 42% girls).	Focus group on gender and cultural-specific messaging/visuals linking tobacco exposure to breast cancer.	Girls preferred messaging that included novel images, personal stories, and ways to avoid secondhand smoke. Boys preferred 'catchy' messages with masculine themes. First Nations and	N/A (not examining a specific intervention)

					Métis participants preferred the use of symbols relevant to their communities.	
**Text message interventions**	**Study design**	**Research Aim**	**Population**	**Intervention/procedure (excludes control conditions)**	**Key Outcomes**	**Authors' Assessment of Gender Inclusion Level Based on Gender Continuum (see Figure 1)**
Crombie et al., 2017 [55]	Randomized control trial	Test the acceptability and feasibility of a brief text message intervention for socioeconomically disadvantaged men who engage in hazardous drinking.	n= 67 Socioeconomically disadvantaged men aged 25 - 44 years who had ≥ 2 episodes of binge drinking.	36 text and multimedia messages sent over 28 days in an interactive motivational interviewing intervention focused on perceptions and reduced drinking.	High retention at follow up (96%). Men reported high levels of satisfaction and enthusiasm with the intervention. Intervention group also showed greater reductions in mean number of binge drinking days in previous 30 days at follow up, though both control and intervention groups showed reductions in all drinking consumption categories except for mean number of moderate drinking days in previous 30	Gender sensitive: the intervention describes some adaptation to intersectional context, but lacks detailed solutions to inequities and harmful norms.

					days (which increased for both groups).	
Irvine et al., 2017 [56]	Randomized control trial	Test the acceptability and feasibility of a text message intervention intended to reduce binge drinking among socioeconomically disadvantaged men.	n= 411 Socioeconomically disadvantaged men aged 25 - 44 years who had ≥ 2 episodes of binge drinking.	112 text messages sent over 12 weeks as an interactive harm reduction approach to elicit alcohol change behaviour.	44% of men in intervention said they had intentions to reduce drinking and 92.4% sent goal-setting responses.	Gender sensitive: the intervention describes some adaptation to intersectional context, but lacks detailed solutions to inequities and harmful norms.
Suffoletto et al., 2016 [57]	Longitudinal study (secondary analysis)	Evaluate a text message alcohol intervention program as a booster to an in-person alcohol intervention mandated for college students.	n= 224 college students (46% women, 54% men).	Interactive PantherTRAC text message program that sent text messages on Thursday and Sunday providing tailored alcohol-related feedback for 6 weeks, aimed at reducing alcohol-related health risks.	90% of text message queries were completed. Men had greater reductions in alcohol consumption when they committed to a drinking-limit goal, a correlation not found for women.	Gender blind: no gender-based considerations are described in the intervention.
